# High-density functional-RNA arrays as a versatile platform for studying RNA-based interactions

**DOI:** 10.1093/nar/gky410

**Published:** 2018-05-28

**Authors:** Jack O Phillips, Louise E Butt, Charlotte A Henderson, Martin Devonshire, Jess Healy, Stuart J Conway, Nicolas Locker, Andrew R Pickford, Helen A Vincent, Anastasia J Callaghan

**Affiliations:** 1 School of Biological Sciences and Institute of Biological and Biomedical Sciences, University of Portsmouth, Portsmouth PO1 2DY, UK; 2 Department of Chemistry, Chemistry Research Laboratory, University of Oxford, Mansfield Road, Oxford OX1 3TA, UK; 3 Faculty of Health and Medical Sciences, School of Biosciences and Medicine, University of Surrey, Guildford GU2 7HX, UK

## Abstract

We are just beginning to unravel the myriad of interactions in which non-coding RNAs participate. The intricate RNA interactome is the foundation of many biological processes, including bacterial virulence and human disease, and represents unexploited resources for the development of potential therapeutic interventions. However, identifying specific associations of a given RNA from the multitude of possible binding partners within the cell requires robust high-throughput systems for their rapid screening. Here, we present the first demonstration of functional-RNA arrays as a novel platform technology designed for the study of such interactions using immobilized, active RNAs. We have generated high-density RNA arrays by an innovative method involving surface-capture of *in vitro* transcribed RNAs. This approach has significant advantages over existing technologies, particularly in its versatility in regards to binding partner character. Indeed, proof-of-principle application of RNA arrays to both RNA–small molecule and RNA–RNA pairings is demonstrated, highlighting their potential as a platform technology for mapping RNA-based networks and for pharmaceutical screening. Furthermore, the simplicity of the method supports greater user-accessibility over currently available technologies. We anticipate that functional-RNA arrays will find broad utility in the expanding field of RNA characterization.

## INTRODUCTION

Understanding the molecular interactions that underlie biological processes is a fundamental aspect of basic biological research and pharmaceutical drug development. The last two decades have seen the development of high-throughput gene expression profiling technologies, including DNA microarrays and RNA-sequencing (RNA-seq), leading to an increased focus on RNA and its interacting partners. Indeed, increasing numbers of RNA–protein (1), RNA–small molecule (e.g. riboswitches (2)) and RNA–RNA interactions (e.g. bacterial small regulatory RNAs (sRNAs; (3)) and eukaryotic microRNAs (miRNA; (4)) are being identified. Of particular interest are the noncoding RNAs (ncRNAs; (5)), such as riboswitches, sRNAs and miRNAs. With emerging roles in bacterial virulence (sRNAs; (6)) and many cancers (miRNAs; (7)), ncRNAs are believed to represent an, as yet, largely untapped resource in the search for novel antimicrobial and/or therapeutic strategies (8–10). In order to unravel the network of interactions in which ncRNAs participate, and realize their potential as pharmaceutical targets, there is a requirement for accessible high-throughput RNA-interaction analysis techniques.

Some progress has been made towards the development of high-density RNA surfaces that are suitable for probing RNA–protein interactions. The Greenleaf (11,12) and Lis (13,14) groups have both utilized the Illumina Next Generation Sequencing platform, capturing transcribed RNAs via a template DNA–protein complex. However, a disadvantage of this method is the complexity of the surface. In particular, the presence of the template DNA precludes the application of this technique to primarily sequence-dependent nucleic acid–RNA interactions typical of most ncRNAs. In contrast, the Smith group (15,16) have employed photolithography, combined with chemical DNA and RNA synthesis, to prime enzymatic synthesis of RNA. The DNA template is removed by DNase treatment resulting in a much simpler surface. However, the use of chemical nucleic acid synthesis limits the length of the RNAs that can be produced. To date, arrays of RNAs only ≤33 nucleotides in length have been generated using this method, which is much shorter than many functional RNAs of biological interest. Furthermore, regardless of the production method, application has been restricted to RNA–protein interactions. Neither of the two approaches have been applied to RNA–small molecule or RNA–RNA interactions. Therefore, while high-density RNA surfaces clearly have potential as a high-throughput RNA-interaction analysis technique, there are also limitations inherent to the currently available production methodologies. Consequently, the need for a high-density functional-RNA platform technology that is capable of broad utility within the RNA field remains.

A major drawback of the existing technologies is the reliance on specialist equipment, not always available to the everyday RNA researcher. Here, we present a simple, innovative method for the production of the first high-density functional-RNA arrays, programmed by DNA arrays, that would be suitable for high-throughput analysis of RNA interactions. We demonstrate proof-of-principle application of these arrays to both RNA–small molecule and RNA–RNA interactions, exemplifying the unique capability of our RNA array platform with regard to the range of binding partners that can be investigated. We highlight the potential utility of RNA arrays as a platform technology in the development of novel antimicrobial strategies and RNA therapies.

## MATERIALS AND METHODS

### Design of DNA *in vitro* transcription templates

**Figure 1.**
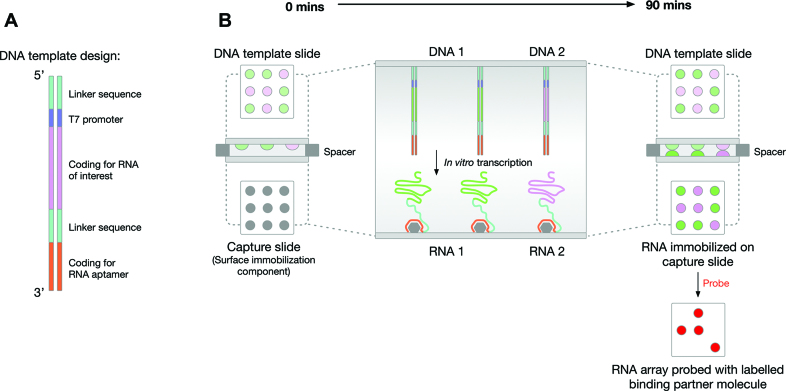
Overview of functional-RNA array production. (**A**) The general design of the DNA *in vitro* transcription templates. From 5′ to 3′ there is: a short, biotinylated linker (to facilitate surface-immobilization of the DNA), a T7 promoter, sequence encoding the RNA of interest, a second linker (to separate the RNA of interest and the RNA aptamer) and sequence encoding an RNA aptamer (to facilitate RNA-capture). (**B**) A schematic representation of the ‘sandwich’ assembly and method used to produce the RNA array. A DNA *in vitro* transcription template array slide is positioned facing an RNA capture slide. Thin spacers are used to physically separate the surfaces. A solution of *in vitro* transcription reagents is inserted between the two surfaces and RNA transcription-capture proceeds for 90 min at 37°C to generate a functional-RNA array. This RNA array can then be used as a platform for investigating RNA-based interactions, e.g. probing with labelled binding partners.

All DNA *in vitro* transcription templates for preparation of DNA template arrays contained a biotinylated 5′ linker (5′–biotin–ctc gag–3′) to facilitate surface-immobilization, immediately followed by a T7 promoter (5′–taa tac gac tca cta tag–3′) and then the sequence encoding the RNA of interest (see Supplementary Table S1 for sequences). DNA templates encoding RNA–aptamer conjugates shared the general design shown in Figure 1A, which includes an additional internal linker sequence (see Supplementary Table S2 for sequences) followed by an aptamer sequence (tobramycin aptamer: 5′–ggc tta gta tag cga ggt tta gct aca ctc gtc gtg agc c–3′; streptavidin aptamer: 5′–acc gac cag aat cat gca agt gcg taa gat agt cgc ggg ccg gg–3′) 3′ to the RNA of interest. The predicted secondary structure of each of the RNA-linker-aptamer conjugates was calculated using Mfold (17) and RNAfold (18) to check that both the RNA of interest and the aptamer were likely to adopt their native structure (see Supplementary Figure S1 as an example).

### Preparation of DNA *in vitro* transcription templates

Initially, non-labelled PCR templates, lacking the biotinylated 5′ linker, were prepared for both unconjugated RNAs and the RNA–aptamer conjugates. PCR templates for unconjugated *rpoS* and the *rpoS*–streptavidin aptamer conjugate were generated by standard PCR using the primers listed in Supplementary Table S3 and plasmid *rpoS*-Blunt II TOPO (19) as the template. The PCR template for the unconjugated malachite green (MG) aptamer was prepared by annealing two complementary single-stranded oligonucleotides (see Supplementary Table S3 for the sequences). All other PCR templates were prepared by gene synthesis using overlapping primers (20; primers are listed in Supplementary Table S3).

DNA *in vitro* transcription templates, containing the biotinylated 5′ linker sequence required for preparing the DNA template arrays, were generated by standard PCR using the PCR templates prepared above, sense primer: 5′–biotin–ctc gag taa tac gac tca cta tag g–3′ and the appropriate antisense primer from Supplementary Table S4.

5′ biotin- and 3′ fluorescently-labelled DNA *in vitro* transcription templates, required for the visualization of the unconjugated MicA and MicA–streptavidin aptamer conjugate (MicA_sa_) DNA template array, were generated by standard PCR using the MicA/MicA_sa_ PCR templates prepared above, sense primer: 5′–biotin–ctc gag taa tac gac tca cta tag g–3′ and antisense primers 5′–Alexa647–aaa agg cca ctc gtg agt–3′ or 5′–Alexa647–gca tgc atc ccg gcc cgc gac tat ctt acg ca–3′ for MicA and MicA_sa_, respectively.

All PCR and *in vitro* transcription templates were purified using a standard spin column-based PCR-clean-up kit and their size was confirmed by agarose gel electrophoresis.

### Preparation of streptavidin surfaces for DNA template arrays

For low-density surfaces, NHS-activated microarray slides (Nexterion H, Schott) were spotted with 10 μl aliquots of 16.7 μM (1 mg/ml) streptavidin in phosphate-buffered saline (PBS; pH 7.4). For high-density surfaces, NHS-activated slides were coated with 90 μl 1μM streptavidin in PBS and covered with a LifterSlip (Thermo Fisher Scientific). Spotted or coated slides were incubated at 37°C for 1 h in a humidified chamber. Where present, LifterSlips were removed and slides were washed, with rocking, once for 5 min with 45 ml PBS containing 0.05% (v/v) Tween 20 (PBS-T), once for 5 min with 45 ml PBS and once for 5 min with 45ml H_2_O (wash-cycle A). They were then submerged in 45 ml 50 mM ethanolamine–HCl pH 8.5 and incubated at room temperature for 30 min, with rocking, to block unreacted NHS functional groups. Wash-cycle A was repeated and the slides were dried by centrifugation at 500 × *g* for 5 min at room temperature.

### Preparation of DNA template arrays

For low-density arrays, between 0.2 μl and 10 μl of 200 nM 5′ biotinylated DNA template in PBS was manually pipetted onto the streptavidin on the prepared streptavidin-spotted slides. For high-density surfaces, an automated arrayer (Genetix Qarray2) fitted with a 200 μm pin-head was used to spot 200 nM 5′ biotinylated DNA template onto prepared streptavidin-coated slides at the spot separations indicated in the Figure legends. The DNA-spotted slides were incubated at 20°C and 50% relative humidity for 30 min, washed using wash-cycle A and dried by centrifugation at 500 × *g* for 5 min at room temperature.

DNA-template arrays containing 3′ fluorescently-labelled MicA–Alexa647 and MicA_sa_–Alexa647 *in vitro* transcription templates were visualized at 670 nm, following excitation at 650 nm, using a microarray slide scanner (aQuire, Genetix or Axon GenePix 4300A, Molecular Devices).

### Preparation of streptavidin RNA capture surfaces

Streptavidin surfaces, for use as RNA capture surfaces, were prepared as described above for DNA template arrays, except that streptavidin was used at a concentration of 16.7 μM (1 mg/ml) in PBS to produce coated (high-density RNA capture) surfaces.

### Preparation of tobramycin RNA capture surfaces

Spotted (low-density) tobramycin RNA capture surfaces were prepared as described above for streptavidin surfaces, except that 5 mM tobramycin in PBS was used instead of streptavidin.

### Production of functional-RNA arrays

The DNA template array, RNA capture surface and *in vitro* transcription mixture (1X Reaction Buffer (MEGAscript T7 Transcription Kit, Thermo Fisher Scientific)), 1X Enzyme Mix (MEGAscript T7 Transcription Kit) and 0.5 mM of ATP, CTP, GTP and UTP) were assembled into a ‘sandwich’ arrangement (Figure 1B), as described below. Two small pieces of Parafilm were placed on top of the RNA capture surface, at each of the short ends of the slide, to act as spacers and prevent the DNA template array and RNA capture surface from coming into direct contact. 150 μl *in vitro* transcription mixture was manually pipetted onto the RNA capture surface and the DNA template array was gently lowered to complete the ‘sandwich’ assembly. This was incubated at 37°C in a humidified chamber for 90 minutes. Following transcription, the slides were separated and the RNA capture surface, now a functional-RNA array, was washed using wash-cycle A and dried by centrifugation at 500 × *g* for 5 min at room temperature.

For preparation of Cy3/Cy5-labelled RNA arrays, the *in vitro* transcription mix was supplemented with 0.05 mM Cy3/Cy5-UTP (GE Healthcare) and the transcription reaction was performed in the dark. Arrays containing Cy3- or Cy5-labelled RNAs were visualized at 570 or 670 nm, following excitation at 550 or 650 nm, respectively, using a microarray slide scanner.

### Preparation of Cy3/Cy5-labelled RNA probes

Internally Cy3/Cy5-labelled *ompA* and Qrr1 RNA probes were generated by *in vitro* transcription. The relevant PCR template (prepared above) was used as the template in an *in vitro* transcription reaction using the MEGAscript T7 Transcription Kit supplemented with 0.05 mM Cy3/Cy5-UTP. The probes were purified using the MEGAclear Transcription Clean-up Kit (Thermo Fisher Scientific).

### Probing functional-RNA arrays

For single probes, 90 μl of 30 nM–3 μM (see Figure legends for experiment-specific details) labelled RNA probe or 20 μM malachite green (Sigma) in hybridization buffer (40 mM Tris (pH 7.8), 6 mM MgCl_2_, 20 mM NaCl) were pipetted over the functional-RNA array. This was covered with a LifterSlip and incubated at 20°C, in a humidified chamber, in the dark for 16 h, unless stated otherwise. The LifterSlip was removed and the probed array was washed with 45 ml hybridization buffer for 1 min and 45 ml water for 10 s (wash-cycle B) before being dried by centrifugation at 500 × *g* for 5 min at room temperature. Simultaneous probing was carried out as described for single probes using concentrations of 3 μM for each individual RNA probe and 20 μM for malachite green. Probed arrays were visualized at 570 nm (Cy3-labelled RNA probes) and/or 670 nm (Cy5-labelled RNA probes and malachite green), following excitation at 550 nm and/or 650 nm, respectively, using a microarray scanner.

## RESULTS

### Strategy for the production of functional-RNA arrays

Our functional-RNA array production method involves the surface-capture of *in vitro* transcribed RNAs. A surface consisting of an array of immobilized double-stranded-DNA *in vitro* transcription templates is positioned opposite to an RNA capture surface, in a ‘sandwich’ arrangement, with the two surfaces connected by a solution of *in vitro* transcription reagents (Figure 1B). To enable the surface-capture of the RNA, each *in vitro* transcription template encodes an RNA aptamer 3′ to the RNA of interest (Figure 1). Following transcription, the aptamer facilitates tethering of the RNA to a capture surface coated with the cognate ligand. The use of RNA aptamers, which rely on adoption of the correct structure for their activity, serves as an internal control for correct folding of the RNA. Furthermore, the 3′ location of the aptamer ensures that only full-length RNAs are immobilized. The physical separation of the DNA template array and the RNA capture surface means that RNA is the only nucleic acid present on the resulting functional-RNA array. This array can then be probed with labelled binding partners (Figure 1B). With each individual spot on the DNA template array capable of encoding a different RNA (e.g. different sequence, different length, different function), it is possible to generate a diverse functional-RNA array suitable for use as a high-throughput platform for RNA-interaction analysis.

### Proof-of-principle functional-RNA array production and applications

Initial proof-of-concept studies to demonstrate (i) surface-capture of *in vitro* transcribed RNAs by RNA aptamers, (ii) functionality of the RNAs on a generated RNA array and (iii) generation of multi-RNA arrays, were performed using manually spotted, low-density, DNA template arrays.

RNA aptamers with nanomolar affinity for small-molecules (e.g. the aminoglycoside tobramycin (21–23)) or proteins (e.g. streptavidin (24)) have been used previously as affinity purification tools (24,25), suggesting that they are an effective means to tether RNA to a solid support. Both tobramycin and streptavidin are commercially available and can be surface-immobilized using standard amine-coupling chemistry to produce the RNA capture surface. Tobramycin, as a small-molecule, would in theory result in a simpler surface in the final RNA array than streptavidin, a protein. Therefore, we first decided to evaluate the use of tobramycin and the tobramycin aptamer.

DNA template arrays were prepared encoding a variety of RNA–tobramycin aptamer (RNA_tob_) conjugates and the corresponding unconjugated RNAs. The RNAs of interest included three bacterial sRNAs and the 5′ UTR of a bacterial mRNA. Following transcription using the ‘sandwich’ arrangement described in Figure 1B, the RNA_tob_ conjugates were preferentially captured on a tobramycin capture surface relative to unconjugated RNAs (Supplementary Figure S2A). However, we also observed non-specific binding of unconjugated RNAs, the extent of which was RNA-dependent (Supplementary Figure S2A).

Due to the non-specific binding of RNA to the tobramycin surface, we next decided to evaluate the use of the streptavidin aptamer for RNA-capture. As above, DNA template arrays were prepared encoding a variety of RNA–streptavidin aptamer (RNA_sa_) conjugates and the corresponding unconjugated RNAs. In this case, the RNAs of interest included bacterial sRNAs, the 5′ UTRs of bacterial mRNAs, and a synthetic RNA aptamer. As shown in Supplementary Figure S2B, following transcription, only the RNA_sa_ conjugates were present on the generated RNA array, the nonconjugated RNAs were not captured. There was no evidence of non-specific binding of RNA to the capture surface. Therefore, we decided to focus our efforts on RNA_sa_ conjugates.

**Figure 2.**
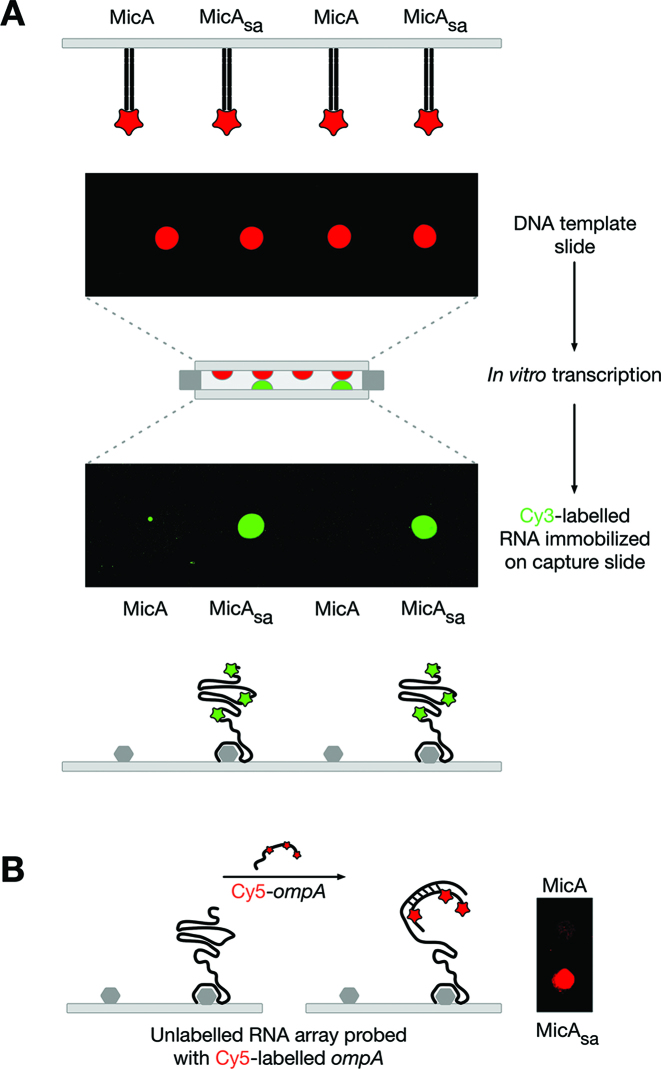
Production of a low-density functional MicA_sa_ RNA array. (**A**) Generation of a Cy3-labelled MicA_sa_ RNA array using a DNA template array of Alexa647-labelled MicA and MicA_sa_. (**B**) A non-labelled MicA_sa_ RNA array (generated as in (A) but without labelling) probed with Cy5-labelled *ompA*.

Having successfully demonstrated the surface-capture of RNA via RNA aptamers we next wanted to demonstrate functionality of the captured RNAs. We selected the well-characterized sRNA MicA–*ompA* mRNA interaction (26; Supplementary Figure S3A), as an example of an RNA–RNA interaction. MicA sRNA regulates the essential gene *ompA* by binding to the 5′ untranslated region (5′ UTR) of *ompA* mRNA, inhibiting translation and targeting the RNA for degradation (26–28). The MicA–*ompA* interaction relies both on the primary RNA sequence of the MicA and *ompA* RNAs and on their secondary structures, namely the accessibility of specific regions (26). A low-density RNA array of MicA_sa_ was generated and probed with *ompA* (Figure 2). As shown in Figure 2B the *ompA* probe binds to the MicA_sa_ RNA array suggesting that the captured-RNA is functional.

**Figure 3.**
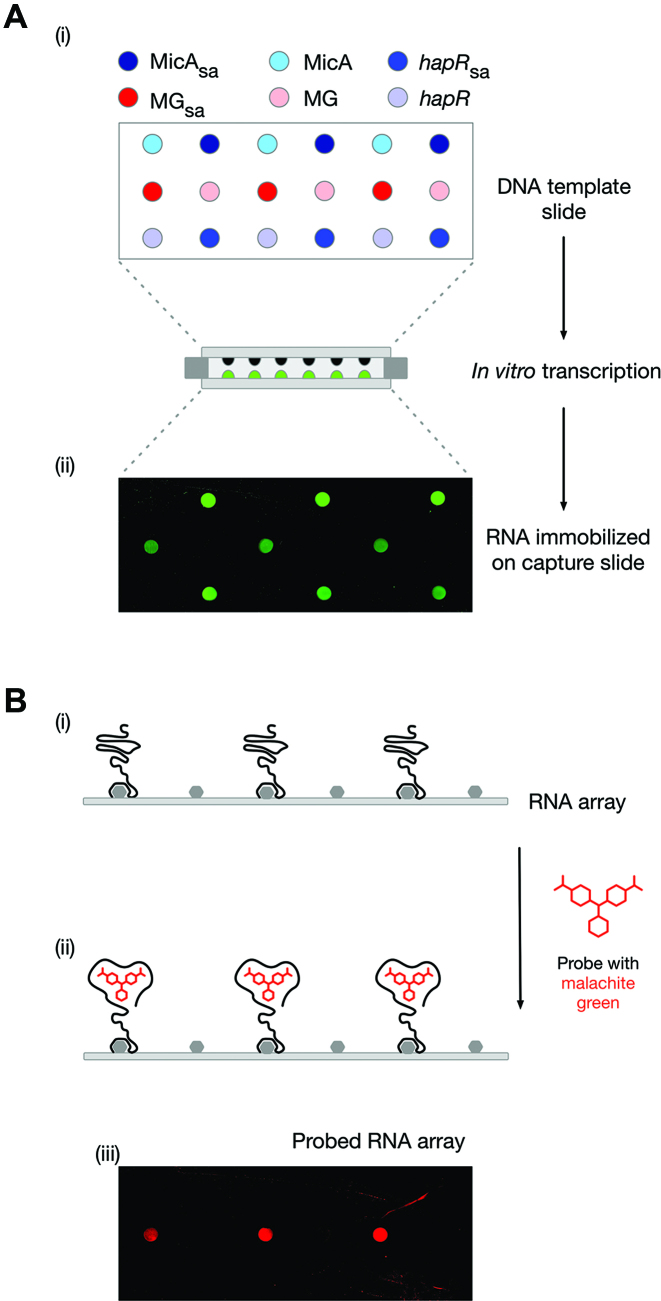
Production of a low-density multi-RNA array. (**A**) Generation of a Cy3-labelled MicA_sa_, *hapR*_sa_ and MG_sa_ RNA array (i) Schematic layout of a DNA template array of MicA, MicA_sa_, *hapR, hapR*_sa_, MG and MG_sa._ (ii) Cy3-labelled RNA array generated from the DNA template array following *in vitro* transcription.(**B**) Probing a low-density multi-RNA array with malachite green. (i) Schematic representation of the middle row of a non-labelled RNA array generated using the DNA template array in A(i). (ii) As in B(i) following probing with malachite green. (iii) A non-labelled MicA_sa_, *hapR*_sa_ and MG_sa_ RNA array (i) (generated as in (A) but without labelling) probed with malachite green. The RNA array images have been mirror imaged so that the spot positions correspond to those shown in the schematics of the DNA template array.

The simple functional-RNA arrays described above have only contained a single RNA species. We next wanted to demonstrate that multi-RNA arrays could be produced using our method. A low-density RNA array of MicA_sa_ sRNA, *hapR*_sa_ (an mRNA involved in virulence of *Vibrio cholera* (29)) and malachite green aptamer (MG_sa_, a synthetic RNA aptamer), was prepared (Figure 3A). The RNA array was probed with the triphenylmethane dye, malachite green (Figure 3B). The specific binding of malachite green to only MG_sa_ indicates that a multi-RNA array has been successfully probed. Furthermore, it demonstrates the functionality of the captured MG_sa_ RNA and application of the functional-RNA array platform to RNA–small molecule interactions.

### Production of a high-density single-RNA array

Our initial validation experiments were carried out using DNA template arrays generated by manual spotting of the DNA *in vitro* transcription templates onto glass slides. Spotting of 10 μl DNA template produces spots of ∼8 mm diameter, allowing for a density of four templates per standard 25 × 75 mm glass slide. By reducing the spotting volume to 0.2 μl, spots of ∼2 mm diameter were produced and a density of 24 templates per slide was achieved. These low- to medium-density arrays may be useful for addressing direct mechanistic questions as part of a basic research project. However, much higher density arrays would be required for most pharmaceutical applications.

**Figure 4.**
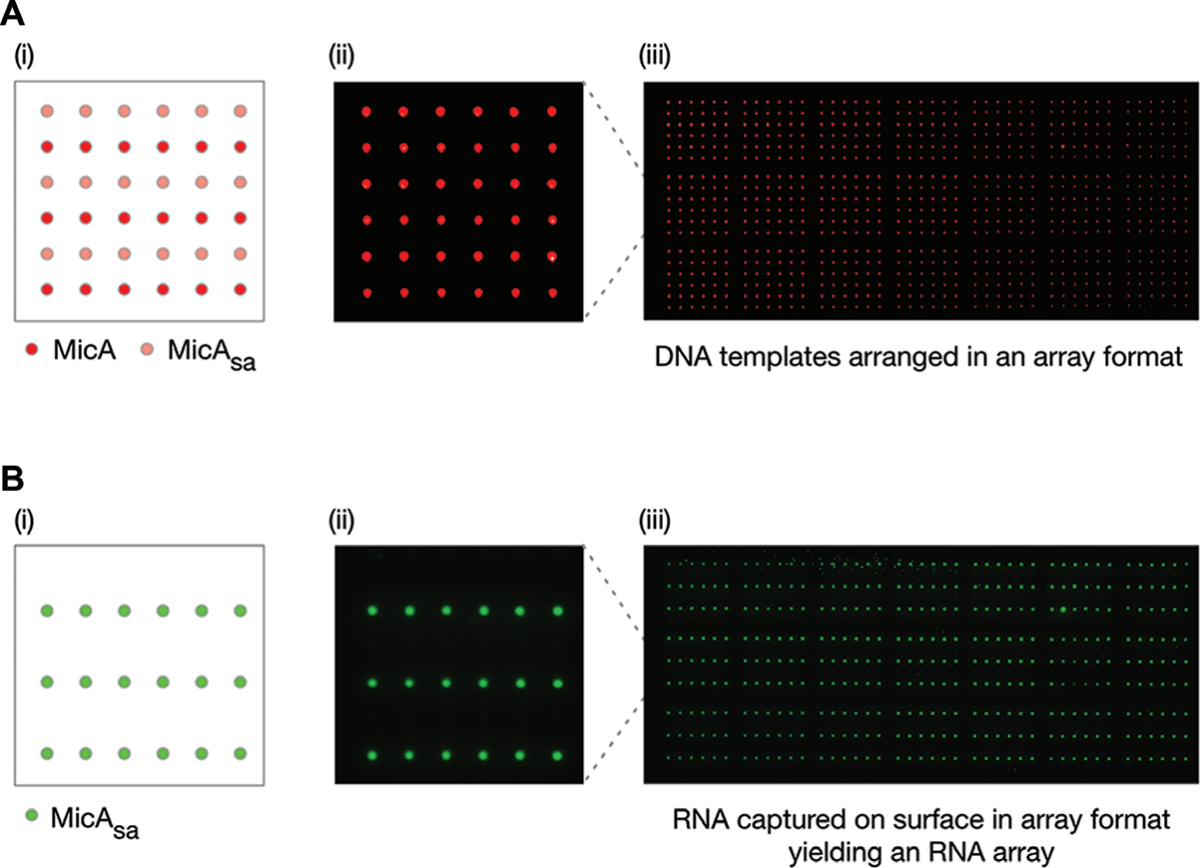
Production of a high-density MicA_sa_ RNA array. (**A**) Preparation of a high-density DNA template array of MicA and MicA_sa_. (i) Schematic layout of a single 6 × 6 grid with alternating rows of MicA and MicA_sa_ templates. (ii) A single 6 × 6 grid of a DNA template array of Alexa647-labelled MicA and MicA_sa_. Individual spots were separated by 750 μm. (iii) The entire DNA template array of 21 repeating 6 × 6 grids as shown in (ii). (**B**) Production of a high-density MicA_sa_ RNA array from the DNA template array described in (A). (i) Schematic of the expected layout of a single grid of the MicA_sa_ RNA array. (ii) A single grid of the Cy3-labelled MicA_sa_ RNA array. (iii) The entire MicA_sa_ RNA array of 21 repeating grids as shown in (ii). The RNA array images have been mirror imaged so that the spot positions correspond to those shown in the schematics of the DNA template array.

To improve the density of the functional-RNA array platform we decided to use an automated arrayer to generate the DNA template arrays. We prepared a DNA template array of 21 identical 6 × 6 grids of alternating rows of MicA and MicA_sa_*in vitro* transcription templates (756 spots in total) (Figure 4A). This was used to produce a high-density MicA_sa_ array (378 spots) (Figure 4B). The spots of captured MicA_sa_ RNA were observed to have an approximately Gaussian profile, presumably resulting from the lateral diffusion of the newly-transcribed RNA from its template DNA spot, before its capture by a streptavidin molecule on the capture surface (Supplementary Figure S4). Further analysis revealed that the 756-spot DNA template array contained individual spots of 76.3 ± 8.2 μm full width at half-maximum (FWHM) (Supplementary Figure S4A) and generated a 378-spot RNA array with spots of 137.1 ± 5.9 μm FWHM (Supplementary Figure S4B). Therefore, the chosen spot-density for the high-density DNA template array was more than sufficient to maintain effective separation of the corresponding RNA spots and generate a high-density RNA array. There is the potential to generate higher-density RNA arrays through optimization of the DNA template array density, reaction conditions, etc. However, this was not the main goal of this work and has not been pursued further.

### Production and applications of high-density multi-RNA arrays

Having successfully generated a high-density RNA array of identical RNA molecules, we wanted to expand our method to the fabrication of high-density multi-RNA arrays. Furthermore, we wanted to demonstrate the potential of these arrays as a high-throughput screening platform for RNA-based interactions.

**Figure 5.**
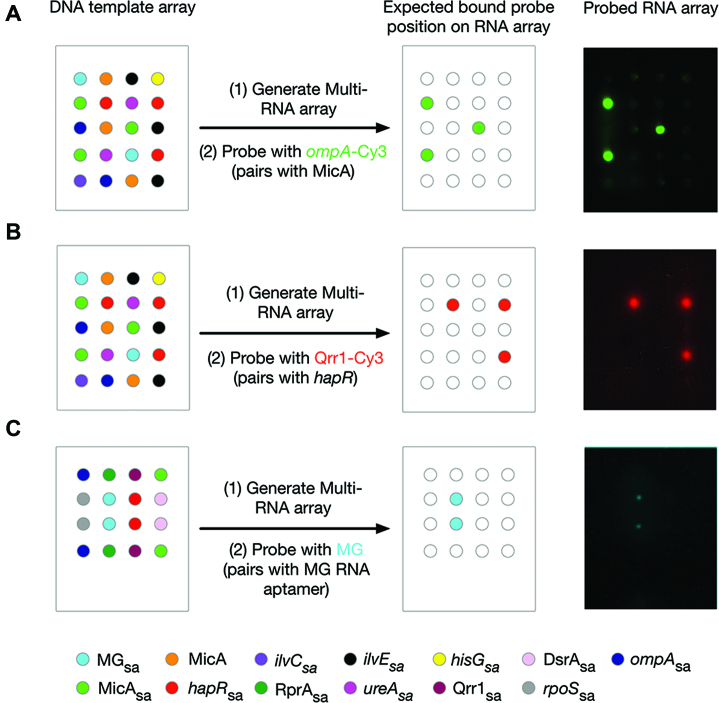
Production and screening of high-density multi-RNA arrays with single probes. (**A**) A DNA template array of MicA_sa_, MicA, *ompA*_sa,_*hapR*_sa_, *ilvC*_sa_, *ilVE*_sa_, *hisG*_sa_, *ureA*_sa_ and MG_sa_ templates (left: schematic of a single-field) was used to generate an eight-RNA array of MicA_sa_, *ompA*_sa,_*hapR*_sa_, *ilvC*_sa_, *ilVE*_sa_, *hisG*_sa_, *ureA*_sa_ and MG_sa_ that was probed with 3 μM Cy3-labelled *ompA* (right). (**B**) A DNA template array of MicA_sa_, MicA, *ompA*_sa,_*hapR*_sa_, *ilvC*_sa_, *ilVE*_sa_, *hisG*_sa_, *ureA*_sa_ and MG_sa_ templates (left: schematic of a single-field) was used to generate an eight-RNA array of MicA_sa_, *ompA*_sa,_*hapR*_sa_, *ilvC*_sa_, *ilVE*_sa_, *hisG*_sa_, *ureA*_sa_ and MG_sa_ that was probed with 2.5 μM Cy3-labelled Qrr1 (right). The probed array has been false-coloured red to match the key. (**C**) A DNA template array of MicA_sa_, Qrr1_sa_, DsrA_sa_, RprA_sa_, *hapR*_sa_, *ompA*_sa_, *rpoS_sa_* and MG_sa_ templates (left: schematic of a single-field) was used to generate an eight-RNA array of MicA_sa_, Qrr1_sa_, DsrA_sa_, RprA_sa_, *hapR*_sa_, *ompA*_sa_, *rpoS_sa_* and MG_sa_ that was probed with 20 μM malachite green (right). The probed array has been false-coloured cyan to match the key. DNA template arrays of repeating 4 × 5 grids (A and B) or 4 × 4 grids (C) were prepared using an automated arrayer with individual spots separated by 1250 μm. Probed RNA images have all been mirror imaged so that the spot positions correspond to those shown in the schematic of the DNA template arrays.

In order to demonstrate utility to RNA–RNA interaction screening, high-density eight-RNA arrays were prepared. These arrays were probed with either MicA-specific *ompA* or Qrr1, an sRNA that binds to and regulates the expression of *hapR* mRNA (30,31; Supplementary Figure S3B). The *ompA* RNA probe correctly discriminated between MicA and seven other RNAs (Figure 5A). Similarly, the Qrr1 probe specifically bound *hapR*_sa_ in the context of seven other RNAs (Figure 5B). In addition, to demonstrate the application of functional-RNA arrays to RNA–small molecule interactions, Figure 5C shows the small molecule malachite green correctly discriminating between MG_sa_ and seven other RNAs.

**Figure 6.**
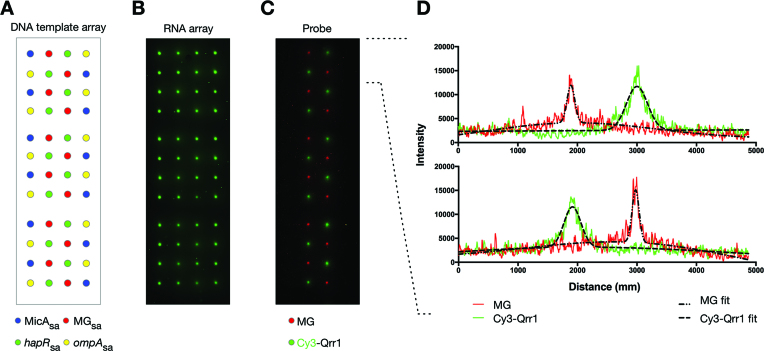
Simultaneous screening of a high-density multi-RNA array with multiple probes. (**A**) Schematic of a DNA template array of repeating 4 × 4 grids of MicA_sa_, *hapR*_sa_, MG_sa_ and *ompA_sa_* templates that was prepared using an automated arrayer with individual spots separated by 1250 μm. (**B**) The generated four-RNA array of Cy3-labelled MicA_sa_, *hapR*_sa_, MG_sa_ and *ompA_sa_*. (**C**) A non-labelled MicA_sa_, *hapR*_sa_, MG_sa_ and *ompA_sa_* RNA array (generated as in (B) but without labelling) probed with 3 μM Cy3-labelled Qrr1 and 20 μM malachite green. The labelled (B) and probed (C) RNA array images have been mirror imaged so that the spot positions correspond to those shown in the schematic of the DNA template array (A). (**D**) Spot profiles for the first two rows of the probed RNA array shown in (**C**). The red and green lines are the spot profile data for malachite green and Qrr1, respectively. The dashed black lines are the least-squares minimization of a function describing the sum of two co-incident Gaussian distributions (one for the main peak, and one for weak signal broadening due to diffusion of non-specifically bound probe):- $f\;( x ) = \mathop \sum \limits_{i = 1}^2 {a_i}\;{\rm{exp}}\left( { - \frac{{{{( {x - {\rm{\mu_i }}} )}^2}}}{{2\sigma _i^2}}}\right)$ where $f( x )$ is the fluorescence intensity at position *x*, and ${a_i},\;{\rm{\mu_i }}$ and ${{\rm{\sigma }}_i}$ are the intensity, centre and standard deviation, respectively, of spot *i*. The FWHM for each spot was calculated as:- $FWHM\; = \;2\sigma \sqrt {2\ln 2}$.

We next attempted simultaneous probing of multi-RNA arrays using multiple probes. Supplementary Figure S5 shows the generation of a RNA array containing MicA_sa_ and *hapR*_sa_, simultaneously probed with two RNAs: *ompA* and Qrr1. In addition to simultaneous screening of multiple RNA–RNA interactions, it is possible to simultaneously screen both RNA–RNA and small molecule–RNA interactions. A high-density four-RNA array containing MicA_sa_, *hapR*_sa_, MG_sa_ and *ompA_sa_* was produced (Figure 6A and B). This was simultaneously probed with Qrr1 and malachite green (Figure 6C). The probed spots measured 151 ± 21 μm and 300 ± 98 μm FWHM, for malachite green and Qrr1 respectively (Figure 6D).

These multi-RNA array experiments are examples of the application of functional-RNA arrays to screening sets of diverse RNAs. However, it may also be useful to screen a series of RNAs that differ by only a single nucleotide. Provided that the nucleotide substitutions occur within the interacting region, using appropriate probing conditions, it should be possible to observe a correlation between the theoretical Gibbs free energy (Δ*G*) of the interaction and the fluorescence intensity of the probe. To demonstrate this, we selected the interaction between *hapR* and Qrr1. The theoretical ΔG of the interaction between the wild-type *hapR* and Qrr1 was calculated using Mfold (17) to be –18.3 kcal/mol (Supplementary Figures S3B and S6A). For a *hapR* mutant, *hapR*(G63C), known to disrupt *hapR*:Qrr1 pairing (31), the theoretical ΔG of the interaction was calculated to be –10 kcal/mol (Supplementary Figure S6A). An RNA array consisting of *hapR*_sa_, *hapR*(G63C)_sa_ and *hapR*(C64G)_sa_, a *hapR* variant resulting in a theoretical *hapR*:Qrr1 ΔG of –14.2 kcal/mol (Supplementary Figure S6A), was generated and probed with Qrr1. As shown in Supplementary Figure S6B, there is a clear correlation between the fluorescence intensity of the Qrr1 probe and the calculated theoretical ΔG for the *hapR*:Qrr1 interaction. Therefore, it is possible to detect single nucleotide substitutions with functional-RNA arrays.

## DISCUSSION

Here we have demonstrated an innovative method for the production of high-density arrays of functional RNAs that involves the surface-capture of *in vitro* transcribed RNAs (Figure 1). Each of the RNAs examined was transcribed from the bacteriophage T7 promoter using a commercially available T7 *in vitro* transcription kit. However, we see no reason why the method could not be adapted for other transcriptions systems, including those of eukaryotic origin. To facilitate surface-capture, an RNA aptamer was conjugated to the 3′ end of the RNA of interest. We evaluated the use of tobramycin and streptavidin aptamers, although other aptamers could be employed. We found that the streptavidin aptamer was the most versatile. For some RNAs, we observed non-specific binding to the tobramycin on the capture surface, a known problem with tobramycin at physiological pH (25). Using this system, we investigated RNAs ranging in size from 38 nucleotides (the malachite green aptamer) to 606 nucleotides (the 5′ UTR of *rpoS* mRNA), with several in the 75–200 nucleotide range (e.g. MicA sRNA: 75 nucleotides; the 5′ UTR of *ompA* mRNA: 167 nt). This size range encompasses many prokaryotic, eukaryotic and viral RNAs of functional interest, as well as biotechnologically relevant synthetic RNAs such as aptamers, and we envisage that longer RNAs could be produced, if required. The functional-RNA arrays were probed with either a small-molecule that fluoresces upon RNA-binding or a fluorescently-labelled RNA binding partner. However, a wide-range of probes could be used, including protein, DNA, RNA and small molecules.

Our production method has significant advantages over previously reported technologies. Firstly, the physical separation of the DNA template and RNA capture surfaces simplifies the final RNA surface, relative to those in the methods based on the Illumina Next Generation Sequencing platform (11–14). The Smith group achieved a simplified surface by nuclease treatment (15,16). However, the use of chemically synthesized *in vitro* transcription templates in their photolithography-based method, limited the size of the RNAs that could be generated. An additional benefit of our method over both of the currently available platforms is the 3′-tethering of the RNA to the capture surface. The conjugation of an aptamer to the 3′ end of the RNA of interest, to facilitate surface-immobilization, ensures that only full-length, correctly folded RNAs are captured.

The advantage of RNA-based tethering, to produce a simpler RNA surface, combined with the ability to immobilize full-length functional RNAs expands the utility of multi-RNA platforms. To-date, applications have focused solely on RNA–protein interactions. The Illumina-based approaches, which retain the DNA template on the ‘RNA’ surface, are unsuitable for studying primarily sequence-driven RNA–RNA interactions and the length-limitations of the photolithography method exclude the investigation of interactions relying on a global structure such as RNA–small molecule interactions. We have now demonstrated the proof-of-principle application of high-density functional-RNA arrays to the investigation of RNA–RNA and RNA–small molecule interactions. Furthermore, our method is more accessible than the existing technologies, both of which require specialist equipment. Preparation of the high-density RNA arrays does require access to an automated arrayer. However, low/medium-density arrays can easily be prepared and probed using standard laboratory equipment.

In demonstrating the application of our functional-RNA arrays to the study of RNA–RNA interactions we have focused on bacterial non-coding small regulatory RNAs (sRNAs). sRNAs act by imperfect base-pairing to an mRNA target, typically within the 5′ UTR, modulating translation initiation and/or transcript stability (3,32). Through this mechanism they regulate genes involved in diverse cellular processes, including bacterial virulence (6). Consequently, sRNA–mRNA interactions are of interest in developing novel antimicrobials (10). Here we have shown that the previously reported MicA–*ompA* (26) and Qrr1–*hapR* (30,31) interactions can be specifically detected using out technology. Both of these interactions are of potential pharmaceutical interest, with *ompA* an essential gene and, therefore, an antimicrobial target (33) and the Qrr1–*hapR* interaction implicated in *V. cholerae* virulence (30,31). We anticipate that the functional-RNA array platform will be used to characterize the complex networks of sRNA–mRNA interactions that exist *in vivo* and in the screening of potential antimicrobial mimics or modulators of these systems.

Although we demonstrate the application of our technology to non-coding bacterial sRNAs, there are equivalent eukaryotic RNA-based systems (miRNA and siRNA (4,34)) that could also benefit from the functional-RNA array platform. For example, miRNAs are key players in cell differentiation, proliferation and survival, and dysregulation of miRNA activity is implicated in many cancers, cardiovascular disease and diabetes (7). Mapping of miRNA networks to identify therapeutic targets would be a potential application of the RNA array platform. Furthermore, antisense oligonucleotides (ASOs) or siRNAs are an attractive strategy for artificially controlling eukaryotic mRNA expression (9). However, efforts to use mRNA-targeting ASOs and siRNAs therapeutically have been hampered by unconvincing/inaccurate data, often the result of off-target effects due to non-specific protein binding or partial complementarity to unintended targets (35). Using the RNA array platform as a screening tool could help to identify and/or circumnavigate potential problems much earlier in the drug-discovery process.

In addition, by using protein synthesis as a read-out, the RNA array platform has scope for use in post-transcriptional regulation assays. These assays would be applicable to the non-coding RNA systems discussed above, small molecule–RNA-based systems (e.g. riboswitches (2)) and the development of synthetic modules based on sRNAs (e.g. toehold switches (36)), or riboswitches (37), which would be of use to the synthetic biology and biotechnology fields. Alternatively, the platform could find utility as a biosensor, particularly for the detection of RNA viruses (38,39).

Overall, we have demonstrated a simple, novel method for the production and application of the first functional-RNA arrays that has significant advantages over previously reported technologies improving accessibility of RNA array platforms to the RNA research community. More importantly, our method is uniquely suitable for the investigation of RNA–RNA and RNA–small molecule interactions. Our RNA arrays have true platform technology capabilities, with broad potential applications in the non-coding RNA, post-transcriptional gene regulation and biosensor fields.

## Supplementary Material

Supplementary Data
