# Supplementary material for: High-density functional-RNA arrays as a versatile platform for studying RNA-based interactions
Source: Nucleic Acids Res. 2018 May 28;46(14):e86. doi: 10.1093/nar/gky410 (PMC6101487; doi:10.1093/nar/gky410)
Supplement: Supplementary Data [file gky410_supplemental_files.pdf]

# Supplementary Material

# High-density functional-RNA arrays as a versatile platform for studying RNA-based interactions

Jack O. Phillips[1†], Louise E. Butt[1†*], Charlotte A. Henderson[1†], Martin Devonshire[1], Jess Healy[2‡], Stuart J. Conway[2], Nicolas Locker[3], Andrew R. Pickford[1], Helen A. Vincent[1*] and Anastasia J. Callaghan[1*]

[1] School of Biological Sciences and Institute of Biological and Biomedical Sciences, University of Portsmouth, Portsmouth, PO1 2DY, UK

[2] Department of Chemistry, Chemistry Research Laboratory, University of Oxford, Mansfield Road, Oxford, OX1 3TA, UK

[3] Faculty of Health and Medical Sciences, School of Biosciences and Medicine, University of Surrey, Guildford, GU2 7HX, UK

[‡] Current address: Department of Pharmaceutical and Biological Chemistry, School of Pharmacy, UCL, 29-39 Brunswick Square, London, WC1N 1AX, UK

* To whom correspondence should be addressed. Tel: +44 (0)23 9284 2055; Fax: +44 (0)23 9284 2070; Email: Anastasia.Callaghan@port.ac.uk. Correspondence may also be addressed to Helen A. Vincent. Tel: +44 (0)23 9284 2055; Fax: +44 (0)23 9284 2070; Email: Helen.Vincent@port.ac.uk. Correspondence may also be addressed to Louise E. Butt. Tel: +44 (0)23 9284 2055; Fax: +44 (0)23 9284 2070; Email: Louise.Butt@port.ac.uk

[†] The authors wish it to be known that, in their opinion, the first three authors should be regarded as joint First Author

## Supplementary References

40. Kerpedjiev,P., Hammer,S. and Hofacker,I.L. (2015) Forna (force-directed RNA): simple and effective online RNA secondary structure diagrams. *Bioinformatics*, **31**, 3377-3379.

41. Henderson,C.A., Vincent,H.A., Stone,C.M., Phillips,J.O., Cary,P.D., Gowers,D.M. and Callaghan,A.J. (2013) Characterization of MicA interactions suggests a potential novel means of gene regulation by small non-coding RNAs. *Nucleic Acids Res*., **41**, 3386-3397.

**Supplementary Table S1. Sequences of the RNAs of interest.** Up to three guanines have been added to the 5' end of each RNA to optimise transcription from the T7 promoter.

| RNA Class | RNA | DNA-template sequence |
|---|---|---|
| sRNAs | MicA | 5'–ggg aaa gac gcg cat ttg tta tca tca tcc ctg aat tca gag atg aaa ttt tgg cca ctc acg agt ggc ctt tt–3' |
| | Stabilised MicA (MS) | 5'–aaa gac gcg cat ttg tta tca tca tcc ctg gga aag cga ggc ttt ccc tgg cca ctc acg agt ggc ctt tt–3' |
| | Qrr1 | 5'–ggt gac ccg caa ggg tca cct agc caa ctg acg ttg tta gtg aat aat caa tgt tca caa ata aca gcc aat aga ctc att cta ttg gct att ttt tt–3' |
| | DsrA | 5'–gga aca cat cag att tcc tgg tgt aac gaa ttt ttt aag tgc ttc ttg ctt aag caa gtt tca tcc cga ccc cct cag ggt cgg gat tt–3' |
| | RprA | 5'–gga cgg tta taa atc aac ata ttg att tat aag cat gga aat ccc ctg agt gaa aca acg aat tgc tgt gtg tag tct ttg ccc atc tcc cac gat ggg ctt ttt ttt–3' |
| mRNAs | *hapR* | 5'–ggc ttt aag tag caa ata aca aaa taa tca tta gag caa aat gct caa tca aca act caa ttg gca agg ata tac ccc tat gga cgc at–3' |
| | *hapR*(G63C) | 5'–ggc ttt aag tag caa ata aca aaa taa tca tta gag caa aat gct caa tca aca act caa ttg cca agg ata tac ccc tat gga cgc at–3' |
| | *hapR*(C64G) | 5'–ggc ttt aag tag caa ata aca aaa taa tca tta gag caa aat gct caa tca aca act caa ttg gga agg ata tac ccc tat gga cgc at–3' |
| | *ompA* | 5'–ggc cag ggg tgc tcg gca taa gcc gaa gat atc ggt aga gtt aat att gag cag atc ccc cgg tga agg att taa ccg tgt tat ctc gtt gga gat att cat ggc gta ttt tgg atg ata acg agg cgc aaa aaa tga aaa aga cag cta tcg cga ttg cag tgg ca–3' |
| | *rpoS* | 5'–ggt tct gag tct tcg ggt gaa cag agt gct aac aaa atg ttg ccg aac aac aag cca act gcg acc acg gtc aca gcg cct gta acg gta cca aca gca agc aca acc gag ccg act gtc agc agt aca tca acc agt acg cct atc tcc acc tgg acct gg ccg act gag ggc aaa gtg atc gaa acc ttt ggc gct tct gag ggg ggc aac aag ggg att gat atc gca ggc agc aaa gga cag gca att atc gcg acc gca gat ggc cgc gtt gtt tat gct ggt aac gcg ctg cgc ggc tac ggt aat ctg att atc atc aaa cat aat gat gat tac ctg agt gcc tac gcc cat aac gac aca atg ctg gtc cgg gaa caa caa gaa gtt aag gcg ggg caa aaa ata gcg acc atg ggt agc acc gga acc agt tca aca cgc ttg cat ttt gaa att cgt tac aag ggg aaa tcc gta aac ccg ctg cgt tat ttg ccg cag cga taa atc ggc gga acc agg ctt ttg ctt gaa tgt tcc gtc aag gga tca cgg gta gga gcc acc tta tga gtc aga–3' |

| | | |
|---|---|---|
| mRNAs | *ilvC* | 5'–ggc ata aca aca taa caa tgt gta aac aca aca tca taa aac aac aca ctc aac atc ata caa taa ata agg aag cac aaa atg gct aac tat ttc aac aca tta aac tta cgt caa aaa tta gac–3' |
| | *ilvE* | 5'–ggt aaa aat tca ttt gta ata cat aac aat aag gac aca aca aca tgg ctt taa aag att tag act ggg cca att tag gct tct cat at–3' |
| | *hisG* | 5'–ggc aaa ata cac aac ctc tcg gaa ggc aac ttt cga gag gtt ttt tat atc cga aat tta aac aca aca tgg gaa aaa caa aat gac aac cac aaa ccg ttt acg tat cgc tct gca gaa gaa agg g–3' |
| | *ureA* | 5'–gga aaa ttt tac aaa cac aaa caa aaa gaa ggt aca aaa tgc att taa ctt caa gag aac aag aaa aac tga tgt tgt ttc tt–3' |
| Synthetic aptamer | Malachite green aptamer (MG) | 5'–gat ccc gac tgg cga gag cca ggt aac gaa tgg atc c–3' |

**Supplementary Table S2. Internal linker sequences for the RNA-aptamer conjugates.**

| Aptamer | RNA of interest | DNA-template sequence |
|---|---|---|
| Tobramycin | MicA | 5'–aca cac aca cac aca cac ac–3' |
| | Stabilised MicA (MS) | 5'–ata tcc ccc ccc ccc ccc cc–3' |
| | Qrr1 | 5'–ttt ttt ttt tcc ccc ccc cc–3' |
| | *hapR* | 5'–aaa aaa aaa aaa aaa aaa a–3' |
| Streptavidin | MicA; Stabilised MicA (MS); Malachite green aptamer (MG); *ilvC*; *ilvE*; *hisG*; *ureA* | 5'–aca cac aca cac aca cac acg cat gca t–(aptamer)–atgcatgc–3' |
| | Qrr1 | 5'–ttt ttt ttt ttt ttt ttt ttt gtg tg–(aptamer)–cac aca–3' |
| | DsrA | 5'–ttt ttt ttt ttt ttt ttt tag ag–(aptamer)–ctc ta–3' |
| | RprA | 5'–ttt ttt ttt ttt ttt ttt gtg tg–(aptamer)–cac ac–3' |
| | *hapR*; *hapR*(G63C); *hapR*(C64G); *ompA* | 5'–aat aat aat aat aat aat aat atg cat gc–(aptamer)–gca tgc at–3' |
| | *rpoS* | 5'–ttt ttt ttt ttt ttt ttt atg cat gc–(aptamer)–gca tgc at–3' |

**Supplementary Table S3. Primer sequences for the preparation of the PCR templates used in generating the DNA *in vitro* transcription templates.** Templates were prepared by gene synthesis from overlapping primers (20), unless indicated by a footnote.

| RNA | Primer | Primer Sequence |
|---|---|---|
| MicA | Sense 1 | 5'–taa tac gac tca cta tag ggg aaa gac gcg cat ttg tta tca tca tcc ctg aat tca gag atg aa–3' |
| | Antisense 1 | 5'–aaa agg cca ctc gtg agt ggc caa aat ttc atc tct gaa ttc agg gat gat gat aac–3' |
| MicA-tobramycin aptamer ($MicA_{tob}$) | Sense 2 | 5'–taa tac gac tca cta tag ggg aaa gac gcg cat ttg tta tca tca tcc ctg aat tca ga–3' |
| | Sense 1 | 5'–tgt tat cat cat ccc tga att cag aga tga aat ttt ggc cac tca cga gtg gcc ttt tac–3' |
| | Antisense 1 | 5'–taa acc tcg cta tac taa gcc gtg tgt gtg tgt gtg tgt gta aaa ggc cac tcg tga gtg–3' |
| | Antisense 2 | 5'–ggc tca gca cga gtg tag cta aac ctc gct ata cta agc cgt–3' |
| MicA-streptavidin aptamer ($MicA_{sa}$) | Sense 3 | 5'–tta tac gac tca cta tag ggg aaa gac gcg cat ttg–3' |
| | Sense 2 | 5'–ggg gaa aga cgc gca ttt gtt atc atc atc cct gaa ttc aga gat gaa att t–3' |
| | Sense 1 | 5'–cat ccc tga att cag aga tga aat ttt ggc cac tca cga gtg gcc ttt tac a–3' |
| | Antisense 1 | 5'–gtc ggt atg cat gcg tgt gtg tgt gtg tgt gtg taa aag gcc act cgt gag t–3' |
| | Antisense 2 | 5'–ccc gcg act atc tta cgc act tgc atg att ctg gtc ggt atg cat gcg tgt g–3' |
| | Antisense 3 | 5'–gca tgc atc ccg gcc cgc gac tat ctt acg ca–3' |
| Stabilised MicA (MS) | Sense 1 | 5'–taa tac gac tca cta tag aaa gac gcg cat ttg tta tca tca tcc ctg gga aag cg–3' |
| | Antisense 1 | 5'–aaa agg cca ctc gtg agt ggc cag gga aag cct cgc ttt ccc agg gat gat gat aac–3' |
| MS-tobramycin aptamer ($MS_{tob}$) | Sense 2 | 5'–taa tac gac tca cta tag aaa gac gcg cat ttg tta tca tca tcc ctg gga aag cg–3' |
| | Sense 1 | 5'–tat cat cat ccc tgg gaa agc gag gct ttc cct ggc cac tca cga gtg gcc ttt tat atc–3' |
| | Antisense 1 | 5'–taa acct cg cta tac taa gcc ggg ggg ggg ggg ggg gat ata aaa ggc cac tcg tga gtg–3' |
| | Antisense 2 | 5'–ggc tca gca cga gtg tag cta aac ctc gct ata cta agc cgg–3' |

| | | |
|---|---|---|
| MS-streptavidin aptamer (MS$_{sa}$) | Sense 3 | 5'–taa tac gac tca cta tag aaa gac gcg ca–3' |
| | Sense 2 | 5'–act cac tat aga aag acg cgc att tgt tat cat cat ccc tgg gaa agc gag g–3' |
| | Sense 1 | 5'–cat ccc tgg gaa agc gag gct ttc cct ggc cac tca cga gtg gcc ttt tac a–3' |
| | Antisense 1 | 5'–gtc ggt atg cat gcg tgt gtg tgt gtg tgt gtg taa aag gcc act cgt gag t–3' |
| | Antisense 2 | 5'–ccc gcg act atc tta cgc act tgc atg att ctg gtc ggt atg cat gcg tgt g–3' |
| | Antisense 3 | 5'–gca tgc atc ccg gcc cgc gac tat ctt acg ca–3' |
| Qrr1 | Sense 2 | 5'–taa tac gac tca cta tag ggt gac ccg caa ggg tca c–3' |
| | Sense 1 | 5'–gtg acc cgc aag ggt cac cta gcc aac tga cgt tgt tag tga ata atc–3' |
| | Antisense 1 | 5'–gaa tga gtc tat tgg ctg tta ttt gtg aac att gat tat tca cta aca acg tca gtt ggc–3' |
| | Antisense 2 | 5'–aaa aaa ata gcc aat aga atg agt cta ttg gct gtt att tgt gaa c–3' |
| Qrr1-tobramycin aptamer (Qrr1$_{tob}$) | Sense 3 | 5'–taa tac gac tca cta tag ggt gac ccg caa g–3' |
| | Sense 2 | 5'–act ata ggg tga ccc gca agg gtc acc tag cca act gac gtt gtt agt gaa taa tc–3' |
| | Sense 1 | 5'–cca act gac gtt gtt agt gaa taa tca atg ttc aca aat aac agc caa tag act ca–3' |
| | Antisense 1 | 5'–gga aaa aaa aaa aaa aaa ata gcc aat aga atg agt cta ttg gct gtt att tgt ga–3' |
| | Antisense 2 | 5'–cct cgc tat act aag ccg ggg ggg ggg aaa aaa aaa aaa aaa aat agc caa tag aa–3' |
| | Antisense 3 | 5'–ggc tca gca cga gtg tag cta aac ctc gct ata cta agc cgg g–3' |
| Qrr1-streptavidin aptamer (Qrr1$_{sa}$) | Sense 3 | 5'–taa tac gac tca cta tag ggt gac ccg c–3' |
| | Sense 2 | 5'–ctc act ata ggg tga ccc gca agg gtc acc tag cca act gac gtt gtt agt gaa taa tca–3' |
| | Sense 1 | 5'–aac tga cgt tgt tag tga ata atc aat gtt cac aaa taa cag cca ata gac tca ttc tat–3' |
| | Antisense 1 | 5'–aaa aaa aaa aaa aaa aaa aaa aaa aaa tag cca ata gaa tga gtc tat tgg ctg tta ttt–3' |
| | Antisense 2 | 5'–act tgc atg att ctg gtc ggt cac aca aaa aaa aaa aaa aaa aaa aaa aaa aaa tag cca–3' |
| | Antisense 3 | 5'–tgt gtg ccc ggc ccg cga cta tct tac gca ctt gca tga ttc tgg tcg g–3' |
| DsrA-streptavidin aptamer (DsrA$_{sa}$) | Sense 3 | 5'– taa tac gac tca cta tag gga aca c–3' |
| | Sense 2 | 5'–aat acg act cac tat agg gaa cac atc aga ttt cct ggt gta acg aat ttt tta a–3' |
| | Sense 1 | 5'–gat ttc ctg gtg taa cga att ttt taa gtg ctt ctt gct taa gca agt ttc atc c–3' |
| | Antisense 1 | 5'–aaa aaa aaa aat ccc gac cct gag ggg gtc ggg atg aaa ctt gct taa gca aga a–3' |
| | Antisense 2 | 5'–gca tga ttc tgg tcg gtc tct aaa aaa aaa aaa aaa aaa aaa atc ccg acc ctg a–3' |
| | Antisense 3 | 5'–tag agc ccg gcc cgc gac tat ctt acg cac ttg cat gat tct ggt cgg tct c–3' |

| | | |
|---|---|---|
| RprA-streptavidin aptamer (RprA$_{sa}$) | Sense 3 | 5’–taa tac gac tca cta tag gga cgg tta taa atc aac–3’ |
| | Sense 2 | 5’–cac tat agg gac ggt tat aaa tca aca tat tga ttt ata agc atg gaa atc ccc tg–3’ |
| | Sense 1 | 5’–gat tta taa gca tgg aaa tcc cct gag tga aac aac gaa ttg ctg tgt gta gtc tt–3’ |
| | Antisense 1 | 5’–aaa aaa aaa aaa agc cca tcg tgg gag atg ggc aaa gac tac aca cag caa ttc gt–3’ |
| | Antisense 2 | 5’–tga ttc tgg tcg gtc aca caa aaa aaa aaa aaa aaa aaa aaa aaa gcc cat cgt gg–3’ |
| | Antisense 3 | 5’–gtg tgc ccg gcc cgc gac tat ctt acg cac ttg cat gat tct ggt cgg tca cac a–3’ |
| *hapR* | Sense 2 | 5’–taa tac gac tca cta tag ggc ttt aag tag caa–3’ |
| | Sense 1 | 5’–ctc act ata ggg ctt taa gta gca aat aac aaa ata atc att aga gca aaa tgc t–3’ |
| | Antisense 1 | 5’–atg cgt cca tag ggg tat atc ctt gcc aat tga gtt gtt ga–3’ |
| | Antisense 2 | 5’–atc ctt gcc aat tga gtt gtt gat tga gca ttt tgc tct aat gat tat ttt gtt a–3’ |
| *hapR*-tobramycin aptamer (*hapR*$_{tob}$) | Sense 3 | 5’–taa tac gac tca cta tag ggc ttt a–3’ |
| | Sense 2 | 5’–taa tac gac tca cta tag ggc ttt aag tag caa ata aca aaa taa tca tta gag caa aat–3’ |
| | Sense 1 | 5’–caa ata aca aaa taa tca tta gag caa aat gct caa tca aca act caa ttg gca agg ata–3’ |
| | Antisense 1 | 5’–ctt ttt ttt ttt ttt ttt tta tgc gtc cat agg ggt ata tcc ttg cca att gag ttg ttg–3’ |
| | Antisense 2 | 5’–cac gag tgt agc taa acc tcg cta tac taa gcc ttt ttt ttt ttt ttt ttt tat gcg tcc–3’ |
| | Antisense 3 | 5’–ggc tca gca cga gtg tag cta aac ctc g–3’ |
| *hapR*-streptavidin aptamer (*hapR*$_{sa}$) | Sense 3 | 5’–taa tac gac tca cta tag ggc ttt aag tag caa ata aca a–3’ |
| | Sense 2 | 5’–tag ggc ttt aag tag caa ata aca aaa taa tca tta gag caa aat gct caa tca a–3’ |
| | Sense 1 | 5’–cat tag agc aaa atg ctc aat caa caa ctc aat tgg caa gga tat acc cct atg g–3’ |
| | Antisense 1 | 5’–cat gca tat tat tat tat tat tat tat tat gcg tcc ata ggg gta tat cct tgc c–3’ |
| | Antisense 2 | 5’–ctt gca tga ttc tgg tcg gtg cat gca tat tat tat tat tat tat tat tat gcg t–3’ |
| | Antisense 3 | 5’–atg cat gcc ccg gcc cgc gac tat ctt acg cac ttg cat gat tct ggt cgg t–3’ |
| *hapR*(G63C)-streptavidin aptamer (*hapR*$_{sa}$) | Sense 3 | 5’–taa tac gac tca cta tag ggc ttt aag tag caa ata aca a–3’ |
| | Sense 2 | 5’–tag ggc ttt aag tag caa ata aca aaa taa tca tta gag caa aat gct caa tca a–3’ |
| | Sense 1 | 5’–cat tag agc aaa atg ctc aat caa caa ctc aat tgc caa gga tat acc cct atg g–3’ |
| | Antisense 1 | 5’–cat gca tat tat tat tat tat tat tat tat gcg tcc ata ggg gta tat cct tgg c–3’ |
| | Antisense 2 | 5’–ctt gca tga ttc tgg tcg gtg cat gca tat tat tat tat tat tat tat tat gcg t–3’ |
| | Antisense 3 | 5’–atg cat gcc ccg gcc cgc gac tat ctt acg cac ttg cat gat tct ggt cgg t–3’ |

| | | |
|---|---|---|
| *hapR*(C64G)-streptavidin aptamer (*hapR*$_{sa}$) | Sense 3 | 5'–taa tac gac tca cta tag ggc ttt aag tag caa ata aca a–3' |
| | Sense 2 | 5'–tag ggc ttt aag tag caa ata aca aaa taa tca tta gag caa aat gct caa tca a–3' |
| | Sense 1 | 5'–cat tag agc aaa atg ctc aat caa caa ctc aat tgg gaa gga tat acc cct atg g–3' |
| | Antisense 1 | 5'–cat gca tat tat tat tat tat tat tat tat gcg tcc ata ggg gta tat cct tcc c–3' |
| | Antisense 2 | 5'–ctt gca tga ttc tgg tcg gtg cat gca tat tat tat tat tat tat tat tat gcg t–3' |
| | Antisense 3 | 5'–atg cat gcc ccg gcc cgc gac tat ctt acg cac ttg cat gat tct ggt cgg t–3' |
| *ompA* | Sense 3 | 5'–taa tac gac tca cta tag ggc cag ggg t–3' |
| | Sense 2 | 5'–ctc act ata ggg cca ggg gtg ctc ggc ata agc cga aga tat cgg tag agt taa tat tga gc–3' |
| | Sense 1 | 5'–ccg aag ata tcg gta gag tta ata ttg agc aga tcc ccc ggt gaa gga ttt aac cgt g–3' |
| | Antisense 1 | 5'–cca aaa tac gcc atg aat atc tcc aac gag ata aca cgg tta aat cct tca ccg gg–3' |
| | Antisense 2 | 5'–gtc ttt ttc att ttt tgc gcc tcg tta tca tcc aaa ata cgc cat gaa tat ctc caa cg–3' |
| | Antisense 3 | 5'–tgc cac tgc aat cgc gat agc tgt ctt ttt cat ttt ttg cgc ctc gtt atc–3' |
| *ompA*-streptavidin aptamer (*ompA*$_{sa}$) | Sense 3 | 5'–taa tac gac tca cta tag ggc cag ggg tgc tcg gca taa gcc gaa gat atc ggt aga–3' |
| | Sense 2 | 5'–cat aag ccg aag ata tcg gta gag tta ata ttg agc aga tcc ccc ggt gaa gga ttt aac cgt–3' |
| | Sense 1 | 5'–ccg gtg aag gat tta acc gtg tta tct cgt tgg aga tat tca tgg cgt att ttg gat gat aac–3' |
| | Antisense 1 | 5'–tgc aat cgc gat agc tgt ctt ttt cat ttt ttg cgc ctc gtt atc atc caa aat acg cca tga–3' |
| | Antisense 2 | 5'–ctg gtc ggt gca tgc ata tta tta tta tta tta tta ttt gcc act gca atc gcg ata gct gtc–3' |
| | Antisense 3 | 5'–atg cat gcc ccg gcc cgc gac tat ctt acg cac ttg cat gat tct ggt cgg tgc atg cat at–3' |
| *rpoS*[a] | Sense 1 | 5'–taa tac gac tca cta tag ggt tct gag tct tcg ggt gaa cag–3' |
| | Antisense 1 | 5'–tct gac tca taa ggt ggc tcc–3' |
| *rpoS*-streptavidin aptamer[b] (*rpoS*$_{sa}$) | Sense 1/2 | 5'–taa tac gac tca cta tag ggt tct gag tct tcg ggt gaa cag–3' |
| | Antisense 1 | 5'–gat tct ggt cgg tgc atg cat aaa aaa aaa aaa aaa aaa tct gac tca taa–3' |
| | Antisense 2 | 5'–atg cat gcc ccg gcc cgc gac tat ctt acg cac ttg cat gat tct ggt cgg–3' |

| | | |
|---|---|---|
| *ilvC*-streptavidin aptamer (*ilvC*$_{sa}$) | Sense 2 | 5'–taa tac gac tca cta tag ggc ata aca aca taa caa tgt gta aac aca aca tca taa aac aac aca ctc aac atc ata caa taa ata agg–3' |
| | Sense 1 | 5'–act caa cat cat aca ata aat aag gaa gca caa aat ggc taa cta ttt caa cac att aaa ctt acg–3' |
| | Antisense 1 | 5'–ctg gtc ggt atg cat gcg tgt gtg tgt gtg tgt gtg tgt cta att ttt gac gta agt tta atg tgt tga aat ag–3' |
| | Antisense 2 | 5'–gca tgc atc ccg gcc cgc gac tat ctt acg cac ttg cat gat tct ggt cgg tat gca tgc gtg–3' |
| *ilvE*-streptavidin aptamer (*ilvE*$_{sa}$) | Sense 2 | 5'–taa tac gac tca cta tag ggt aaa aat tca ttt gta ata cat aac aat aag gac aca aca aca tgg ctt taa aag att tag act g–3' |
| | Sense 1 | 5'–cat ggc ttt aaa aga ttt aga ctg ggc caa ttt agg ctt ctc ata tac aca cac aca cac aca cac gca tgc ata ccg acc ag–3' |
| | Antisense 1/2 | 5'–gca tgc atc ccg gcc cgc gac tat ctt acg cac ttg cat gat tct ggt cgg tat gca tgc gtg–3' |
| *hisG*-streptavidin aptamer (*hisG*$_{sa}$) | Sense 2 | 5'–taa tac gac tca cta tag ggc aaa ata cac aac ctc tcg gaa ggc aac ttt cga gag gtt ttt tat atc cga aat tta aac aca aca tgg–3' |
| | Sense 1 | 5'–ccg aaa ttt aaa cac aac atg gga aaa aca aaa tga caa cca caa acc gtt tac gta tcg ctc tgc ag–3' |
| | Antisense 1 | 5'–ctg gtc ggt atg cat gcg tgt gtg tgt gtg tgt gtg tcc ctt tct tct gca gag cga tac gta aac g–3' |
| | Antisense 2 | 5'–gca tgc atc ccg gcc cgc gac tat ctt acg cac ttg cat gat tct ggt cgg tat gca tgc gtg–3' |
| *ureA*-streptavidin aptamer (*ureA*$_{sa}$) | Sense 2 | 5'–taa tac gac tca cta tag gga aaa ttt tac aaa cac aaa caa aaa gaa ggt aca aaa tgc att taa ctt caa gag aac aag–3' |
| | Sense 1 | 5'–gca ttt aac ttc aag aga aca aga aaa act gat gtt gtt tct tac aca cac aca cac aca cac gca tgc ata ccg acc ag–3' |
| | Antisense 1/2 | 5'–gca tgc atc ccg gcc cgc gac tat ctt acg cac ttg cat gat tct ggt cgg tat gca tgc gtg–3' |
| Malachite green aptamer (MG)[c] | Sense 1 | 5'–taa tac gac tca cta tag gat ccc gac tgg cga gag cca ggt aac gaa tgg atc c–3' |
| | Antisense 1 | 5'–gga tcc att cgt tac ctg gct ctc gcc agt cgg gat cct ata gtg agt cgt att a–3' |
| MG-streptavidin aptamer (MG$_{sa}$) | Sense 2 | 5'–taa tac gac tca cta tag gat ccc gac tgg cga gag cca ggt aac gaa tgg atc–3' |
| | Sense 1 | 5'–gag cca ggt aac gaa tgg atc cac aca cac aca cac aca cac gca tgc ata ccg a–3' |
| | Antisense 1 | 5'–cgg ccc gcg act atc tta cgc act tgc atg att ctg gtc ggt atg cat gcg tgt g–3' |
| | Antisense 2 | 5'–gca tgc atc ccg gcc cgc gac tat ctt–3' |

[a]Generated by standard PCR using plasmid *rpoS*-Blunt II TOPO (19) as the template.

[b]Generated by two consecutive standard PCR reactions. The first utilised plasmid *rpoS*-Blunt II TOPO (19) as the template and the Sense 1/2 and Antisense 1 primers. The product from this PCR was used as the template in a second PCR with the Sense 1/2 and Antisense 2 primers. [c]Generated by annealing the complementary oligonucleotides.

**Supplementary Table S4. Anti-sense primer sequences for the preparation of the DNA *in vitro* transcription templates from the PCR templates.**

| RNA | Primer Sequence |
|---|---|
| MicA | 5'–aaa agg cca ctc gtg agt ggc caa aat ttc atc tct gaa ttc agg gat gat gat aac–3' |
| $MicA_{tob}$ | 5'–ggc tca gca cga gtg tag cta aac ctc gct ata cta agc cgt–3' |
| $MicA_{sa}$ | 5'–gca tgc atc ccg gcc cgc gac tat ctt acg ca–3' |
| Stabilised MicA (MS) | 5'–aaa agg cca ctc gtg agt ggc cag gga aag cct cgc ttt ccc agg gat gat gat aac–3' |
| $MS_{tob}$ | 5'–ggc tca gca cga gtg tag cta aac ctc gct ata cta agc cgg–3' |
| $MS_{sa}$ | 5'–gca tgc atc ccg gcc cgc gac tat ctt acg ca–3' |
| Qrr1 | 5'–aaa aaa ata gcc aat aga atg agt cta ttg gct gtt att tgt gaa c–3' |
| $Qrr1_{tob}$ | 5'–ggc tca gca cga gtg tag cta aac ctc gct ata cta agc cgg g–3' |
| $Qrr1_{sa}$ | 5'–tgt gtg ccc ggc ccg cga cta tct tac gca ctt gca tga ttc tgg tcg g–3' |
| $DsrA_{sa}$ | 5'–tag agc ccg gcc cgc gac tat ctt acg cac ttg cat gat tct ggt cgg tct c–3' |
| $RprA_{sa}$ | 5'–gtg tgc ccg gcc cgc gac tat ctt acg cac ttg cat gat tct ggt cgg tca cac a–3' |
| *hapR* | 5'–atc ctt gcc aat tga gtt gtt gat tga gca ttt tgc tct aat gat tat ttt gtt a–3' |
| $\textit{hapR}_{tob}$ | 5'–ggc tca gca cga gtg tag cta aac ctc g–3' |
| $\textit{hapR}_{sa}$ | 5'–atg cat gcc ccg gcc cgc gac tat ctt acg cac ttg cat gat tct ggt cgg t–3' |
| *ompA* | 5'–tgc cac tgc aat cgc gat agc tgt ctt ttt cat ttt ttg cgc ctc gtt atc–3' |
| $\textit{ompA}_{sa}$ | 5'–atg cat gcc ccg gcc cgc gac tat ctt acg cac ttg cat gat tct ggt cgg tgc atg cat at–3' |
| *rpoS* | 5'–tct gac tca taa ggt ggc tcc–3' |
| $\textit{rpoS}_{sa}$ | 5'–atg cat gcc ccg gcc cgc gac tat ctt acg cac ttg cat gat tct ggt cgg–3' |
| $\textit{ilvC}_{sa}$ | 5'–gca tgc atc ccg gcc cgc gac tat ctt acg cac ttg cat gat tct ggt cgg tat gca tgc gtg–3' |
| $\textit{ilvE}_{sa}$ | 5'–gca tgc atc ccg gcc cgc gac tat ctt acg cac ttg cat gat tct ggt cgg tat gca tgc gtg–3' |
| $\textit{hisG}_{sa}$ | 5'–gca tgc atc ccg gcc cgc gac tat ctt acg cac ttg cat gat tct ggt cgg tat gca tgc gtg–3' |
| $\textit{ureA}_{sa}$ | 5'–gca tgc atc ccg gcc cgc gac tat ctt acg cac ttg cat gat tct ggt cgg tat gca tgc gtg–3' |
| Malachite green aptamer (MG) | 5'–gga tcc att cgt tac ctg gct ctc gcc agt cgg gat cct ata gtg agt cgt att a–3' |
| $MG_{sa}$ | 5'–gca tgc atc ccg gcc cgc gac tat ctt–3' |

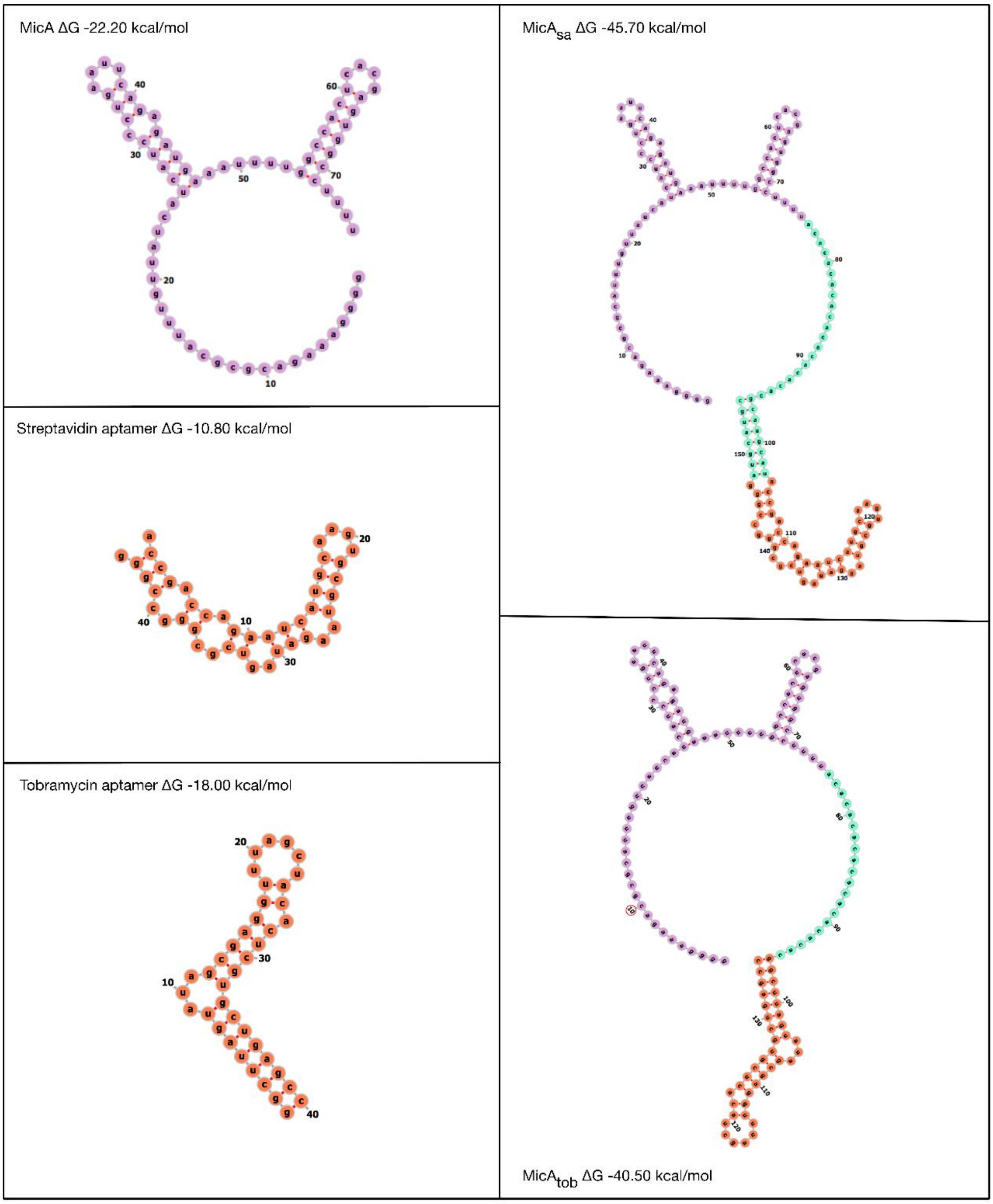


**Supplementary Figure S1. Design of MicA-aptamer conjugate RNAs.** The RNA prediction programs Mfold (17) and RNAfold (18) were utilised to design MicA-linker-aptamer conjugate RNAs that would allow both MicA and the aptamers to adopt their native structures. Structures were predicted for unconjugated MicA, streptavidin aptamer and tobramycin aptamer and the $MicA_{sa}$ and $MicA_{tob}$ conjugates. Structures were visualised using forna (40) with MicA shown in purple, the aptamers in orange and the linker in green. The predictions were evaluated by comparison to the published structures for MicA (26) and the

streptavidin (24) or tobramycin aptamer (21-23), as appropriate. The final design for $MicA_{sa}$ was 5’-MicA-$(AC)_{10}$GCAUGCAU-streptavidin aptamer-AUGCAUGC-3’ and the final design for $MicA_{tob}$ was 5’-MicA-$(AC)_{10}$-tobramycin aptamer-3’.

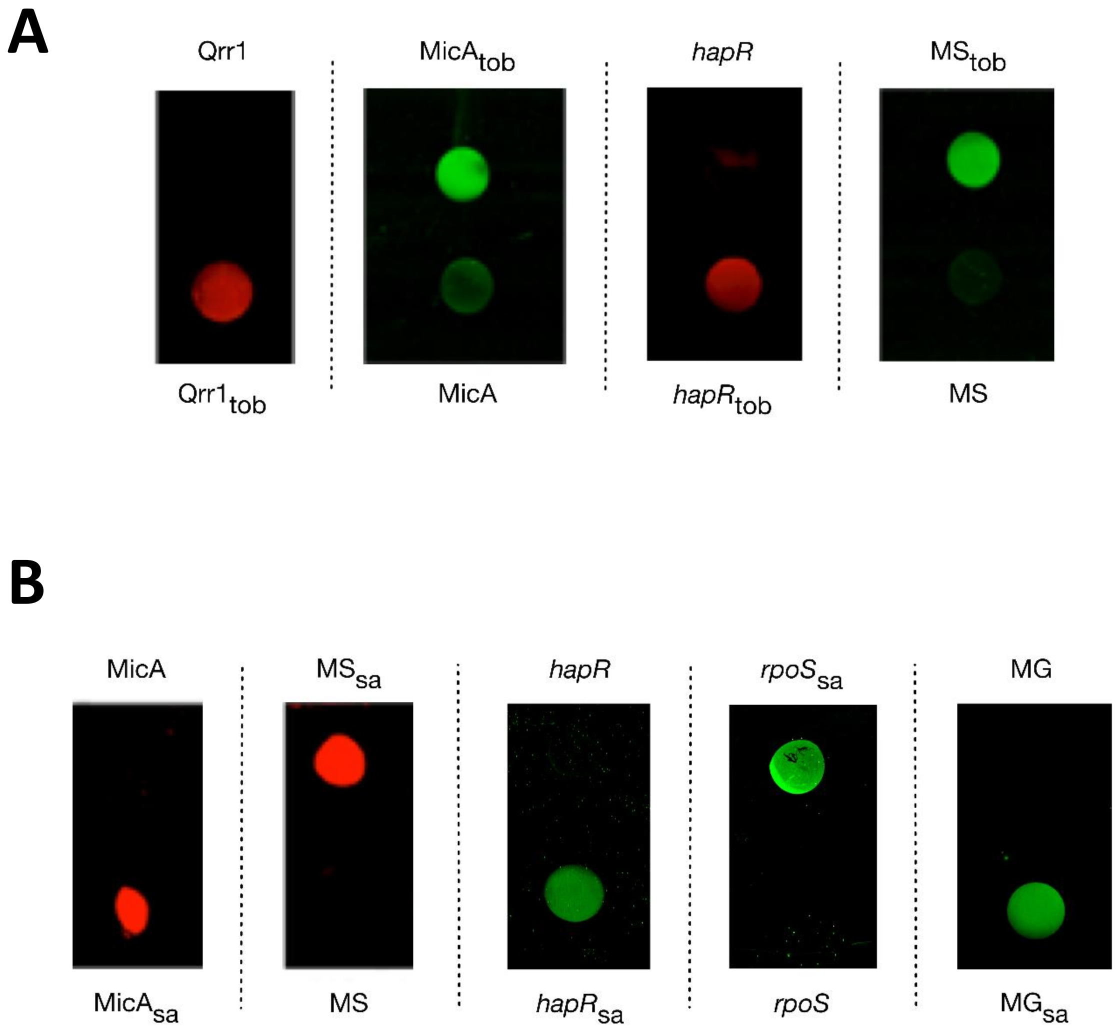


**Supplementary Figure S2. Surface-capture of RNA using RNA aptamers.** (A) RNA arrays using the tobramycin aptamer. RNA arrays for unconjugated and tobramycin aptamer conjugates of sRNAs (MicA, MS (a modified form of MicA (41)) and Qrr1) and the 5’ UTR of *hapR* mRNA. MicA, $MicA_{tob}$, MS, and $MS_{tob}$ were labelled with Cy3 and Qrr1, $Qrr1_{tob}$, *hapR* and $hapR_{tob}$ were labelled with Cy5. (B) RNA arrays using the streptavidin aptamer. RNA arrays for unconjugated and streptavidin aptamer conjugates of sRNAs (MicA, MS), the 5’ UTRs of *hapR* and *rpoS* mRNAs, and a synthetic RNA aptamer that binds malachite green (MG). MicA, $MicA_{sa}$, MS, and $MS_{sa}$ were labelled with Cy5 and *hapR*, $hapR_{sa}$, *rpoS*, $rpoS_{sa}$, MG and $MG_{sa}$ were labelled with Cy3.

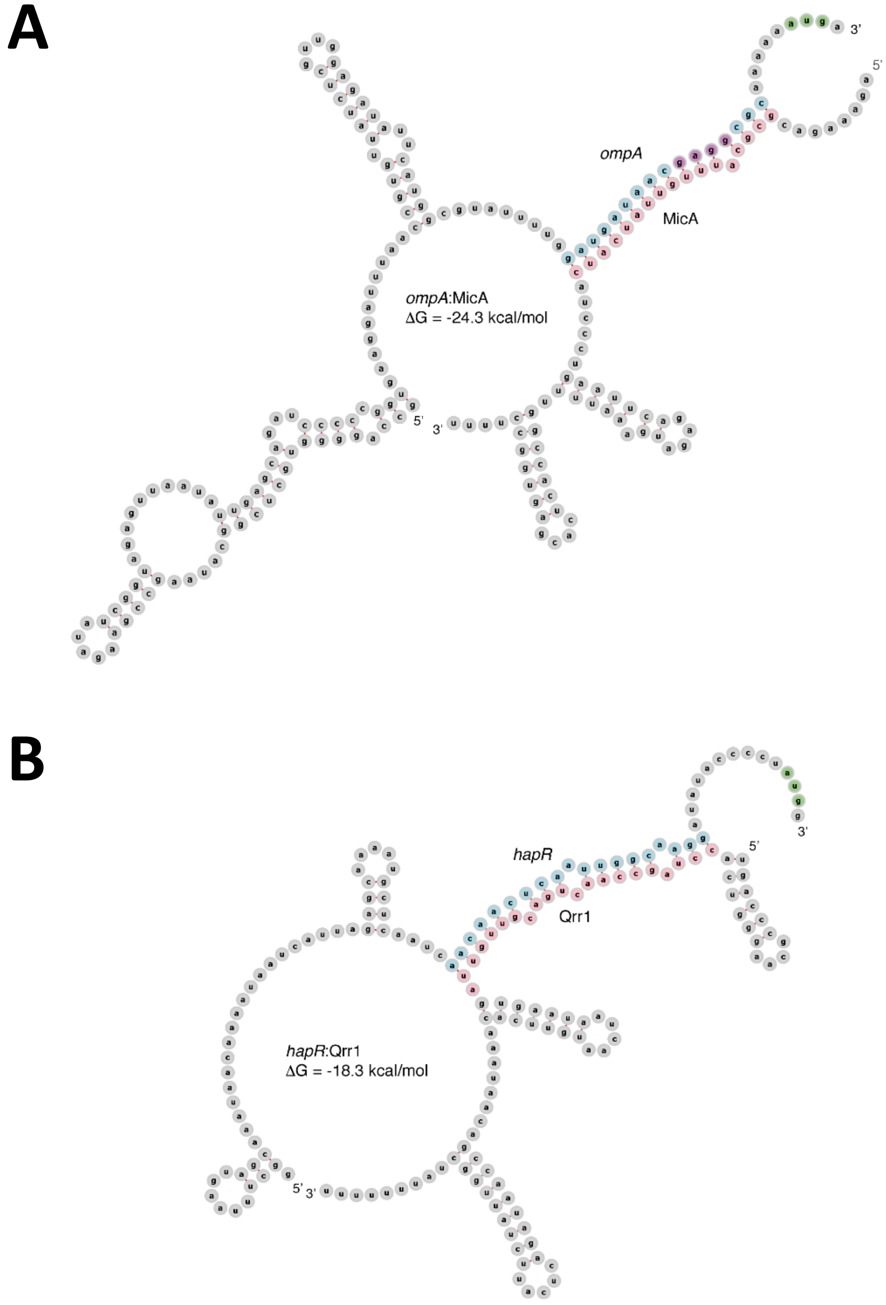


**Supplementary Figure S3. Interactions between sRNAs and their mRNA targets.** RNA secondary structures showing (A) the *ompA*:MicA and (B) the *hapR*:Qrr1 interaction. The structures are based on published interactions (26,30,31) and visualised using forna (40). The interacting region is shown in blue for the mRNA and pink for the sRNA. The Shine-Dalgarno sequence for *ompA* is shown in purple and the start codons for *ompA* and *hapR* are shown in green. The Gibbs free energy for the interacting region was calculated using Mfold (17).

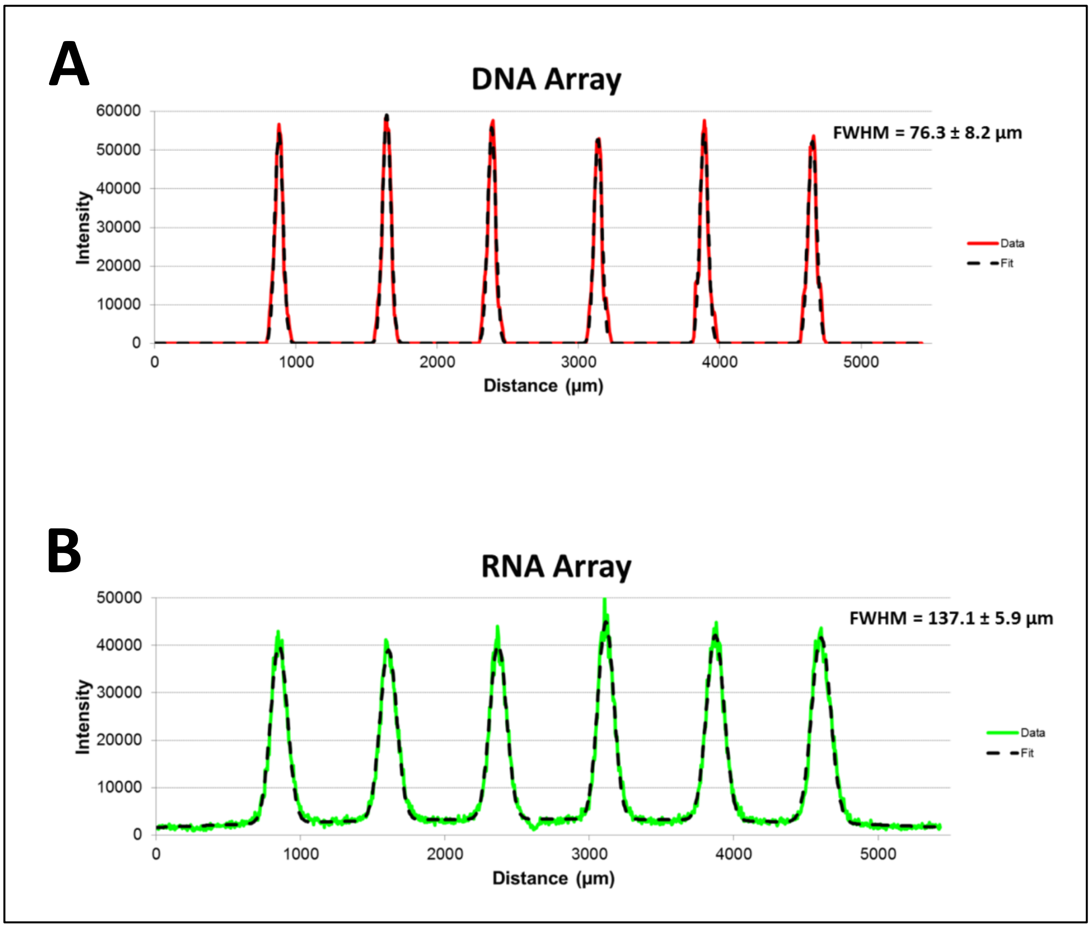


**Supplementary Figure S4. Spot profiles of a high-density DNA template array and the corresponding high-density single-RNA array.** (A) Spot profiles for the second row of the high-density DNA template array grid shown in Figure 4A(ii). The red line represents the spot profile data. The dashed black line is the least-squares minimisation of a function describing the sum of six Gaussian distributions (one for each peak) with a single quadratic baseline correction:-

$$f(x) = \sum_{i=1}^{6} a_i \exp\left(-\frac{(x-\mu_i)^2}{2\sigma_i^2}\right) - (mx^2 + bx + c)$$

where $f(x)$ is the fluorescence intensity at position *x*, and $a_i$, $\mu_i$ and $\sigma_i$ are the intensity, centre and standard deviation, respectively, of spot *i*. The full-width at half maximum (FWHM) for each spot was calculated as:-

$$FWHM = 2\sigma\sqrt{2\ln 2}$$

(B) Spot profiles for the first row of the high-density $MicA_{sa}$ RNA array grid shown in Figure 4B(ii). The green line represents the spot profile data. The dashed black line is the least-squares minimisation of the function described in part (A) above. The FWHM was calculated as in part (A) above.

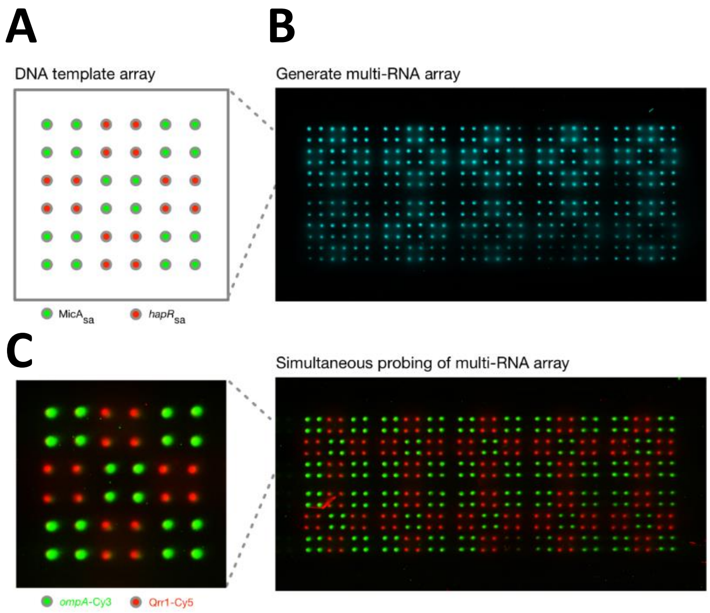


**Supplementary Figure S5. Simultaneous screening a high-density multi-RNA array with multiple RNA probes.** (A) Schematic of a single 6 x 6 grid of a DNA template array of MicA$_{sa}$ and *hapR*$_{sa}$ templates that was prepared using an automated arrayer with individual spots separated by 750 µm. (B) The generated two-RNA array of 10 repeating 6 x 6 grids of Cy3-labelled MicA$_{sa}$ and *hapR*$_{sa}$. (C) A non-labelled MicA$_{sa}$ and *hapR*$_{sa}$ RNA array (generated as in (B) but without labelling) probed with 3 µM Cy3-labelled *ompA* and 3 µM Cy5-labelled Qrr1. The labelled (B) and probed (C) RNA array images have been mirror imaged so that the spot positions correspond to those shown in the schematic of the DNA template array (A).

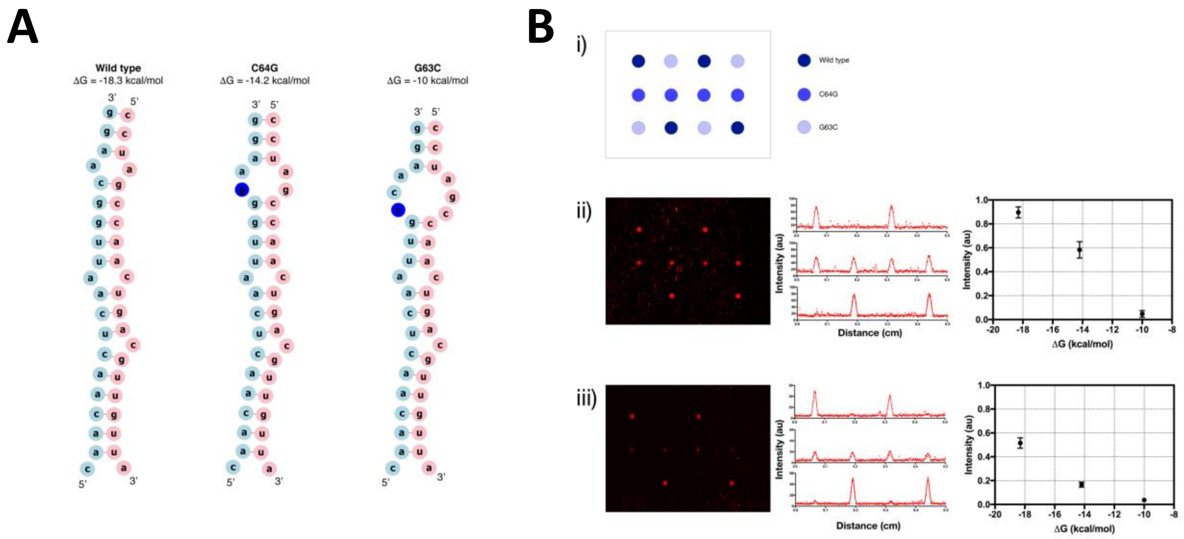


**Supplementary Figure 6. Correlation between fluorescence intensity and Gibbs free energy**. (A) Interacting region between three *hapR* variants (wild type, C64G and G63C) and Qrr1 visualised using forna (40). *hapR* is shown in pale blue, with the variant nucleotides in dark blue, and Qrr1 is shown in pink. The predicted structures and theoretical Gibbs free energy (ΔG) for the interactions were calculated using Mfold (17). (B) An RNA capture surface was prepared using 1 µM streptavidin. *hapR*$_{sa}$ (wild type), *hapR*(G63C)$_{sa}$ and *hapR*(C64G)$_{sa}$ were generated by *in vitro* transcription and diluted to 200 nM in hybridisation buffer. A three-RNA array of *hapR*$_{sa}$ (wild type), *hapR*(G63C)$_{sa}$ (G63C) and *hapR*(C64G)$_{sa}$ (C64G), arranged in 4 x 3 grids with individual spots separated by 1250 µm, was prepared in duplicate using an automated arrayer ((i) schematic of a single-field of the RNA array). The arrays were incubated at room temperature, in a humidified chamber, for 30 minutes. They were washed using wash-cycle A and dried by centrifugation at 500 x *g* for 5 minutes, at room temperature. Each array was probed for 1 hour at room temperature with Cy5-labelled Qrr1 at either (ii) 300 nM or (iii) 30 nM. The spot intensity profiles for each row of the arrays were fit using the least-squares minimisation of a function describing the sum of four Gaussian distributions:

$$f(x) = \sum_{i=1}^{4} a_i \exp\left(-\frac{(x-\mu_i)^2}{2\sigma_i^2}\right)$$

where $f(x)$ is the fluorescence intensity at position $x$, and $a_i$, $\mu_i$ and $\sigma_i$ are the intensity, centre and standard deviation, respectively, of spot $i$ ((ii) and (iii), middle). Mean fluorescence intensity for the spots corresponding to each *hapR* variant was plotted against the calculated theoretical ∆G for the respective *hapR* variant:Qrr1 interaction ((ii) and (iii), right).
